# Does Medical Students’ Personality Traits Influence Their Attitudes toward Medical Errors?

**DOI:** 10.3390/healthcare6030101

**Published:** 2018-08-17

**Authors:** Chia-Lun Lo, Hsiao-Ting Tseng, Chi-Hua Chen

**Affiliations:** 1 Department of Health Business Administration, Fooyin University, Daliao District, Kaohsiung 831, Taiwan; allenlo.tw@gmail.com; 2 Department of Information Management, Tatung University, Zhongshan District, Taipei 104, Taiwan; 3 College of Mathematics and Computer Science, Fuzhou University, Minhou County, Fuzhou 350100, China; chihua0826@gmail.com

**Keywords:** medical errors, personality, attitude, medical students

## Abstract

This study examined medical students’ perceptions towards medical errors and the policy of the hospital within the internship curriculum, and explored how aspects of personality traits of medical students relate to their attitude toward medical errors. Based on the theory of the Five-Factor-Model (FFM) and related literature review, this study adopted a self-devised structured questionnaire to distribute to 493 medical students in years five to seven in the top three medical schools, representing a 56.7% valid questionnaire response rate. Results showed that agreeableness is more important than other personality traits, and medical students with high agreeableness are good communicators and have a more positive attitude to avoid errors in the future. On the contrary, students with low neuroticism tended to be more relaxed and gentle. If medical educators can recruit new students with high agreeableness, these students will be more likely to effectively improve the quality of medical care and enhance patient safety. This study anticipates that this method could be easily translated to nearly every medical department entry examination, particularly with regards to a consciousness-based education of future physicians.

## 1. Background

Patients receive health care to improve their health. However, since the increase of medical error events, medical errors have caused media coverage and public concern about patient safety. Medical errors will greatly affect the safety of patients. Hence, how to reduce the severity and frequency of medical errors has become an important issue in fields related to medical care. The teaching material known as Tomorrow’s Doctors [1] emphasizes that improving communication between physicians and patients may reduce medical errors. Usherwood [2] empirically showed that communication skills can have positive effects on students’ learning of listening skills and compassion; such skills can also improve physician-patient relationships and patient satisfaction [3]. Therefore, worldwide healthcare institutions emphasize that clinicians can reduce the probability of medical errors by improving their communication with patients. Meanwhile, more and more studies [4,5,6,7,8,9,10,11,12,13,14,15,16] highlight the importance of attitude toward medical errors in medical students’ education.

Physician training takes a considerable amount of time. Medical students, especially those in the later years of medical school, should be taught good communication skills to reduce the obstacles in physician-patient relationships during their education. Internship systems were expected to increase physicians’ efficiency and value attached to patient safety [17]. To reach these goals, Taiwan launched a comprehensive medical education reform in 2002. Educators in Taiwan adopted multiple approaches—interviews, personality tests, entrance examinations, etc.—to identify new medical students with both high academic ability and suitable personalities for clinical work, and help them become qualified physicians in the future, so as to further reduce medical errors.

The National Patient Safety Foundation (NPSF) proposed that helping medical school students understand medical errors can help them avoid similar mistakes [18]. Lester and Tritter [19] also mentioned that training medical students in communication skills could improve their future behavior as physicians. Seiden [20] suggested that medical students play important roles in the prevention of medical errors, and that teaching them to understand possible medical errors can effectively enhance patient safety. However, although suitable personality traits and good communication skills in medical students have been valued by healthcare institutions in various countries, medical students are often neglected in studies on the improvement of patient safety education [20]. Studies pointed out that most physicians agree that the reduction of medical errors is an important issue, but the number of medical errors actually reported is much lower than expected [10]. This is because most medical education programs attach importance to practical, professional techniques, and do not focus on educating students about medical errors. Flin et al. [21] investigated medical students’ attitude towards medical errors and found that most students had insufficient knowledge about how to report errors. Muller [16] proposed that irreversible medical errors might make interns experience a loss of self-confidence or self-esteem, and feel guilt or other negative emotions. Fischer et al. [22], using resident doctors as subjects, found that subjects’ personality traits affected how they reported errors and learned. Lievens [23] found that in past studies, when people have more positive and cheerful personality traits, their attitudes towards learning outcomes, self-efficacy, behaviors, performance, etc., are more positive. Other research has also pointed out that when medical staff members have higher self-efficiency for avoiding medical errors, they are less likely to make such errors [24]. Self-efficiency is defined as staff members’ belief in reducing errors and enhancing patient safety [25]. In other words, when medical staff have positive, strong feelings of confidence about their own roles and possess attitudes towards avoiding errors and being competent, they will avoid making errors.

In the past, relevant research pointed out that the younger the doctor, the more likely they are to make medical errors [26]. However, this does not mean that the occurrence of medical errors is a necessary route in the process of medical service development. The National Patient Safety Foundation (NPSF) has said that educating medical students about medical errors can help future physicians avoid similar mistakes [18]. Seiden [20] also believes that medical students play an important role in the prevention of medical errors; educating them to understand medical errors can effectively improve patient safety. In the face of medical errors, it is very important to have a positive attitude of learning from mistakes. For example, in the aviation industry and the energy industry, for example, employees are encouraged to report on possible errors and adverse events, and learn from mistakes to avoid making similar mistakes in the future [27,28]. There is a lack of research on medical errors in medical students in Taiwan, and we have no way of knowing what attitudes and ideas they have. However, attitudes will affect behavior. After a period of time, this group of medical students will be put into the workplace and become important members of the medical industry. Therefore, it is necessary to understand their attitudes toward medical errors.

As seen in the above research, physicians’ personality traits may affect their attitude towards facing medical errors, and it is very important that they have appropriate concepts of and attitudes towards patient safety. For medical educators, the purpose of medical education is to cultivate future medical personnels’ ability to grasp the overall picture of their patient’s health. The Harvard College of Medicine considers the cultivation of an appropriate personality and the ability to make effective value judgments to be the most important educational outcomes (or aims of education). If this study is able to teach medical students how to view and understand medical errors, this study would likely be able to correct their misconceptions, which is especially important for medical students about to become interns in hospitals. Therefore, identifying the appropriate personality traits for being an efficient medical student is one of the important factors of the reform and improvement of current medical education. However, this study does not know how various personality traits influence medical students’ responses to medical errors. Therefore, this study examined the relationship between personality traits and attitudes toward medical errors in a sample of students with internship experience from their final years of medical school. The results could inform the recruitment of future medical students.

## 2. Methods

### 2.1. Participants

A questionnaire survey was sent to 866 medical students (in their 5th, 6th and 7th years), from three medical schools in Taiwan. This study chose this group for the survey because in Taiwan, medical students in their final three years must attend practical training in hospitals. In the end, there were total of 493 (56.9%) valid questionnaires. Demographic data was also collected to obtain accurate backgrounds for all of the participating students. Among them, there were 343 males (71.0%) and 140 females (29.0%); most of the respondents were 21 to 25 years old, with 272 (56.8%); followed by 198 (41.3%) from 26 to 30 years old, and seven (1.5%) from 31 to 35 years old. There were two (1.7%) over 36 years old and four respondents who did not fill in this field. The average age of respondents was 24.66 years old, with a standard deviation of 1.67. The youngest respondents were 22 years old, and the oldest were 38 years old. There were 224 (46.7%) in fifth year, 207 (43.1%) in sixth year, and 49 (10.2%) in seventh year. There were three respondents who did not fill out this field.

The research was approved by the dean of each department at each medical school, and after compiling a list of all students, this study sent the questionnaire via email. Students were asked to complete the questionnaire and send it back to us if they were interested in taking part in the study.

### 2.2. Instruments

The study used a cross-sectional design, and the questionnaire had three sections. The first and second sections were based on a review of the medical error literature, and questions employed a six-point Likert-like scale, ranging from not at all agree (1) to agree completely (6). Except for the personality section, the main sections included the following items:

#### 2.2.1. First Section: Attitudes Towards Medical Errors

- Disclosing and reporting medical errors covered respondents’ attitudes towards disclosing and reporting medical errors after they have occurred (five items).
- Reacting to and learning about medical errors measured respondents’ attitudes towards how they react to and learn about medical errors (four items).
- Emotional reaction covers respondents’ emotional reactions to errors after they have occurred (four items).
- Self-efficacy measured respondents’ level of self-awareness and perceived competence in avoiding medical errors (four items).
- Safety promotion covered respondents’ attitudes towards promoting safety (three items).
- Self-ability asked respondents to rate how well they believe they can do their job (three items).

#### 2.2.2. Second Section: Factors Affect the Occurrence of Medical Errors

- Training and communication asked respondents to indicate their level of agreement with employee training and the effectiveness of communication for avoiding medical errors (six items).
- Management system also measured respondents’ degree of recognition for avoiding medical errors (six items).

A factor analysis was conducted to determine whether student responses were in line with the themes outlined above. In addition, this study measured respondents’ personality traits according to the Five-Factor-Model (FFM). The FFM is a model for describing human personality, which proposes that various personality characteristics can be grouped under five higher-order personality domains: Extraversion, Agreeableness, Conscientiousness, Emotional Stability, and Openness [29]. The research tool contains 43 items employing a six-point Likert scale ranging from 1 (strongly disagree) to 6 (strongly agree). Focus groups and a pilot study was carried out using 15 interns. Demographic data was also covered in the questionnaire. The internal consistency in the pilot study was acceptable (Cronbach’s α > 0.7).

### 2.3. Data Analysis

This study first excluded invalid questionnaires, and used descriptive statistics, including mean, to represent a central location where the results were relatively concentrated. Standard deviation (SD) was used to represent the degree of discreteness of the results. This study then examined how differences in the personality traits of medical students related to differences in their attitudes towards medical errors. In addition to the Pearson correlation coefficient, this study used multiple regression analyses to test the relationship between personality traits and attitudes towards medical errors.

## 3. Results

### 3.1. Experimental Environments

The questionnaires were distributed to 866 medical students with internship experience, and 493 valid questionnaires were received across the three years (valid response rate = 56.9%). The goodness of fit test of the questionnaires showed significant differences. The final sample contained 213 respondents in medical school A, 113 in medical school B, and 167 in medical school C, as shown in Table 1.

Seventy-one percent of respondents were male, and the mean (SD) age was 24.66 (1.67) years old. Four hundred and sixty-one (96%) respondents had interned in medical centers, and among them, 126 (26.7%) had been in the surgical department, 11 (23.5%) in internal medicine, 77 (16.3%) in the gynecological department, 70 (14.8%) in pediatrics, and 88 (18.6%) respondents in the remaining departments including X-rays, family medicine, otolaryngology (ENT), neurology, psychiatry, etc.

The personality traits investigated in this study were Extraversion, Openness, Conscientiousness, Agreeableness, and Neuroticism. The relationships between these dimensions and students’ attitudes towards medical errors are summarized in Table 2.

Medical students tended to have high Conscientiousness (4.18, SD = 0.75) and Agreeableness (4.16, SD = 0.59), but low Neuroticism (3.58, SD = 0.64). Regarding attitudes towards medical errors, most showed high scores on reacting to and learning about medical errors (6.64, SD = 1.01), while they tended to have lower scores on disclosing and reporting medical errors (5.23, SD = 1.20) and self-ability (4.93, SD = 1.22). For the factors that might affect the occurrence of medical errors, students agreed significantly more with training and communication (7.02, SD = 0.89) compared with management systems (6.39, SD = 1.00).

Regarding to the relationships between personality traits and attitudes toward medical errors, the results show that disclosing and reporting medical errors showed significant positive correlations with the personality traits of Extraversion, Openness, and Conscientiousness; reacting to and learning about medical errors showed significant positive correlations with Extraversion, Openness, Conscientiousness, and Agreeableness; emotional reaction showed a significant positive correlation with only Agreeableness; self-efficiency showed a significant positive correlation with Agreeableness, and a significant negative correlation with Neuroticism; and finally, self-ability showed a significant positive correlation with Extraversion, Openness, Conscientiousness, and Agreeableness. Regarding the factors that might affect the occurrence of medical errors, both training and communication and management systems had significant positive correlations with Extraversion, Openness, Conscientiousness, and Agreeableness, while training and communication also showed a significant negative correlation with Neuroticism.

### 3.2. Comparison of Personality Traits with High Identification and Attitudes Towards Medical Errors

This study determined the standard deviations of the average values of students’ personality traits to denote which traits students showed high or low identification with, and analyzed how the personality traits that students highly identified with influenced their attitudes towards medical errors, as shown in Table 3.

For disclosing and reporting medical errors, the gender of medical students could have explained the statistically significant differences in variance between personality traits and attitudes towards medical errors (F = 4.454, *p* < 0.001). The coefficient of determination was R^2^ = 0.074, adjusted R^2^ = 0.073. After controlling for gender using the regression coefficient test, this study found a significant relationship between Conscientiousness and disclosing and reporting medical errors (β = 0.178, *p* < 0.05), indicating that respondents with higher Conscientiousness may also have more positive attitudes towards disclosing and reporting medical errors.

For reacting to and learning about medical errors, gender could again account for statistically significant differences (F = 5.991, *p* < 0.001). The coefficient of determination was R^2^ = 0.096, adjusted R^2^ = 0.093. After controlling for gender, this study found a statistically significant relationship between Agreeableness (β = 0.197, *p* < 0.01) and reacting to and learning about medical errors.

For emotional reaction, gender could explain statistically significant differences (F = 2.560, *p* < 0.05). The coefficient of determination was R^2^ = 0.043, adjusted R^2^ = 0.032. After controlling for gender, this study found no statistically significant relationship between personality traits and emotional reaction.

For self-efficiency, gender could explain statistically significant differences (F = 12.114, *p* < 0.001). The coefficient of determination was R^2^ = 0.177, adjusted R^2^ = 0.160. After controlling for gender, this study found a statistically significant relationship between Conscientiousness (β = 0.267, *p* < 0.001) and self-efficiency.

For safety promotion, gender could explain the statistically significant difference. After controlling for gender, this study found a statistically significant relationship between Agreeableness (β = −0.145, *p* < 0.05) and safety promotion.

For self-ability, gender could explain statistically significant differences (F = 7.839, *p* < 0.001). The coefficient of determination was R^2^ = 0.121, adjusted R^2^ = 0.118. After controlling for gender, this study found that Extraversion (β = 0.138, *p* < 0.05) and Agreeableness (β = 0.216, *p* < 0.001) had statistically significant relationships with self-ability.

For training and communication, gender could explain the statistically significant difference (F = 10.960, *p* < 0.001). The coefficient of determination was R^2^ = 0.163, adjusted R^2^ = 0.154. After controlling for gender, this study found that Extraversion (β = 0.233, *p* < 0.01) and Agreeableness (β = 0.234, *p* < 0.001) had statistically significant relationships with training and communication.

Finally, for management systems, genders could again explain statistically significant differences (F = 8.282, *p* < 0.001). The coefficient of determination was R^2^ = 0.163, adjusted R^2^ = 0.123. After controlling for gender, this study found a statistically significant relationship between Agreeableness (β = 0.136, *p* < 0.001) and management systems.

## 4. Discussions

### 4.1. Experiment Result Discussions

The training process that medical students undergo is very strict, and after they graduate, they must obtain a medical license in order to become fully-fledged medical practitioners. Despite this, physicians seem unable to cope with or resolve medical errors even when they often encounter them. Mizahi [30] found that when hospital staff members made a medical error, they typically adopted three coping strategies: denial, discounting, and distancing. Medical students’ learning attitudes typically develop during their internship, passed down by supervising physicians. If medical school students are given an inaccurate understanding of the disclosure of medical errors and have unsuitable personality traits, they are more likely to conceal medical errors. Previous studies on this issue have discussed its fundamental causes, including notification platforms [31,32], disclosure [33,34], personality traits or gender [16], and emotions [33]. This study discussed the basic personality dimensions, attempted to identify the attitudes of interning medical students toward medical errors, and examined the influences of personality traits on these attitudes.

The results of this study showed that medical students scored highest on Agreeableness, followed by Conscientiousness, Openness, and Extraversion; they scored the lowest on Neuroticism. These differed from the results of Lievens et al.’s [23] study on medical students, which also used the FFM; Vohra et al. [25] pointed out that effective medical students often have personality traits of Agreeableness or Extraversion according to the well-known “Big Five” Inventory (Five Factor Model (FFM)). Moreover, these researchers found that students scored highest on Extraversion, followed by Agreeableness. These differences related to the educational system in Taiwan. Lievens et al. [23] found that Conscientiousness might affect medical students’ grades on their written examinations before graduation; however, medical students in Taiwan enrolled in universities according to their grades on written examinations, and thus most of them are relatively diligent and hardworking. This study can see from the various characteristics of these personality traits [35] that high Agreeableness should be helpful for medical students in communicating with patients and cooperating with other hospital staff; in addition, the cautiousness, sense of responsibility, and drive that accompanies individuals with high Conscientiousness would be helpful for avoiding medical errors.

The majority of interns believe that medical errors are avoidable, and that training can effectively reduce the occurrence of these errors [36]. This study arrived at a similar conclusion: nearly 70% of respondents believed errors to be avoidable, and more than half regarded training and education to be the most important factors that affect medical errors. Furthermore, more than 90% of respondents attached importance to training and communication. According to Singh et al. [37], among the common medical errors made by interns, 70% are caused by miscommunication with team members and 58% are due to a lack of technical expertise and knowledge. Thus, the likelihood that interns would make a medical error due to a lack of education or miscommunication with team members is very high.

Active disclosure of possible errors might help patients and their family members understand an error situation and reduce the occurrence of litigations [38]. Gallagher [39] divided medical errors into three categories: near-miss, relatively not serious, and relatively serious. When medical errors are relatively serious, physicians tend to support error disclosure. While the present study was only concerned with categorizing errors according to whether they caused injury, the results were similar to those of Gallagher and colleagues in that when errors caused injury, students tended to view disclosure more positively than when errors did not cause injury. In this study, the emotional reaction attitude dimension was found to be the most negatively viewed, with around 90% of respondents having worry that being accused might affect their future employment; this is similar to the findings of Muller and Ornstein’s [16] study, wherein they discussed the issues that resident physicians and interns tend to worry about after errors occur.

This study found that Agreeableness and Conscientiousness have the greatest influence on medical students’ attitude towards medical errors. Students with high Agreeableness tend to pay more attention to organization management and systems for avoiding errors, and have more positive attitudes toward personnel communication and coping with medical errors; these features may be determined by their high empathy. Medical students with high Conscientiousness are more likely to be confident in avoiding errors and have a greater sense of justice, often wanting to disclose the error after they have made it. Compared with other personality traits, Agreeableness is more likely to have a significant influence on feelings of responsibility towards and learning of errors, promotion of safety, self-ability, training and communication, and the health care management system.

Previous studies investigated the attitudes of medical students towards medical errors and patient safety, but only those of first-year medical students. Thus, the samples provided less knowledge about medical errors and how physicians report them [21]. The subjects of this study were medical students in their final three years, who had already entered hospitals for internships, and the results were generally positive, as in Flin’s [21] study. However, this study provides more comprehensive evidence showing that medical students who have high Agreeableness tend to have more positive attitudes toward medical errors in general.

### 4.2. Study Limitations

This study faces the following research limitations. Firstly, this study chose medical students who were in their 5th, 6th, or 7th year as subjects, and while the overall response rate was acceptable, there were fewer students in their 7th year. This may be because 7th year students are too busy with their internships, and spend less time in school, which would lead to a lower response rate. In addition, because samples were taken from only three hospitals, it is not clear whether these results can be extrapolated to the entire medical student population.

Secondly, the main problem with this research is to understand the personality traits of medical students and their attitudes towards medical errors. Hopefully, effective interventions can be put in place in the medical education process to reduce the incidence of medical errors. However, medical errors are sometimes caused by poor medical communication, but this study has not discussed medical communication. In addition, there may be individual biases in the personality traits of medical students. In other words, there may be systematic errors in communication between physicians, medical students, and support teams, mainly because of the lack of openness.

## 5. Conclusions and Future Work

From the results of the present study and those of previous studies, it is clear that medical students with high Agreeableness have more positive attitudes towards medical errors, and are more likely to avoid errors in the future; in addition, in encountering such errors, they will be well equipped to face and solve them. Finally, if medical educators can recruit new students with high Agreeableness, these students will be more likely to effectively improve the quality of medical care and enhance patient safety. Therefore, the results of this study can inform the future recruitment of medical school students. Further research is required with larger sample sizes, including other medical personnel or subjects from other academic backgrounds. By comparing these other groups, this study will be able to further generalize the results.

## Figures and Tables

**Table 1:** Survey response rate.

School	Total	Surveys Completed	Response Rate
“A” medical school	447	213	47.7%
“B” medical school	128	113	88.3%
“C” medical school	291	163	57.4%
Grand total	866	493	56.9%

**Table 2:** Means, standard deviations and correlation matrices for combined data with *n* = 493.

Dimensions		Scale (Number of Items)	Mean	SD	a1	a2	a3	a4	a5	b1	b2	b3	b4	b5	b6	b7	b8
Personality traits	a1	Extraversion	3.94	0.67	−												
	a2	Openness	4.17	0.74	0.75 ***	−											
	a3	Conscientiousness	4.18	0.75	0.70 ***	0.72 ***	−										
	a4	Agreeableness	4.61	0.59	0.55 ***	0.59 ***	0.58 ***	−									
	a5	Neuroticism	3.58	0.64	−0.16 ***	−0.26 ***	−0.27 ***	−0.23 ***	−								
Medical students’ attitude towards medical errors	b1	Disclosing and reporting medical errors (5)	5.23	1.20	0.19 ***	0.20 ***	0.26 ***	0.22 ***	−0.06	−							
	b2	Reacting to and learning about medical errors (4)	6.64	1.01	0.25 ***	0.17 ***	0.24 ***	0.41 ***	−0.09	0.34 ***	−						
	b3	Emotional reaction (4)	5.96	1.10	0.06	0.01	0.02	0.11 *	0.07	−0.02	0.18 ***	−					
	b4	Self-efficiency (4)	4.81	1.24	0.39 ***	0.41 ***	0.40 ***	0.29 ***	−0.15 **	0.33 ***	0.03	−0.08	−				
	b5	Safety promotion (3)	6.24	1.22	−0.09 *	−0.07	−0.07	−0.17 ***	−0.04	−0.12 *	−0.30 ***	−0.25 ***	0	−			
	b6	Self-ability (3)	4.93	1.22	0.26 ***	0.27 ***	0.26 ***	0.29 ***	−0.06	0.25 ***	0.10 *	−0.01	0.36 ***	−0.09	−		
	b7	Training and communication (6)	7.02	0.89	0.30 ***	0.19 ***	0.19 ***	0.41 ***	−0.12 **	0.12 **	0.55 ***	0.30 ***	0.06	−0.29 ***	0.09 *	−	
	b8	Management systems (6)	6.39	1.00	0.35 ***	0.31 ***	0.26 ***	0.33 ***	−0.08	0.18 ***	0.35 ***	0.17 ***	0.17 ***	−0.19 ***	0.14 **	0.69 ***	−

*: *p* < 0.05; **: *p* < 0.01; ***: *p* < 0.001; SD = Standard Deviation

**Table 3:** Regression between the attitude of medical error and medical students’ personality traits for combined data with *n* = 493.

Variables	Disclosing and Reporting Medical Errors		Reacting to and Learning about Medical Errors		Emotional Reaction		Self-Efficiency		Safety Promotion		Self-Ability		Training and Communication		Management Systems	
	β	t-Value	β	t-Value	β	t-Value	β	t-Value	β	t-Value	β	t-Value	β	t-Value	β	t-Value
Gender	0.032	0.59	−0.041	0−.775	0.120	2.224 *	0.135	2.680 **	−0.032	−0.584	−0.065	−1.246	−0.075	−1.474	−0.076	−1.471
Openness	−0.031	−0.424	0.127	1.764	−0.056	−0.763	0.169	2.495 *	0.019	0.257	0.138	1.973 *	2.330	3.313 **	0.128	1.831
Extraversion	0.067	0.908	−0.015	0−.202	−0.076	−1.016	−0.045	−0.648	−0.058	−0.761	0.023	0.322	−0.070	−0.990	0.140	1.942
Conscientiousness	0.178	2.496 *	0.045	0.642	0.026	0.360	0.267	3.995 ***	−0.021	−0.297	0.057	0.828	0.013	0.196	0.024	0.340
Agreeableness	0.072	1.145	0.197	3.216 **	−0.104	−1.662	0.068	1.155	−0.145	−2.296 *	0.216	3.550 ***	0.234	3.972 ***	0.136	2.268 ***
Neuroticism	−0.060	−1.103	−0.033	0−0.602	−0.092	−1.677	−0.007	−0.14	−0.083	−1.499	0.080	1.500	−0.080	−1.53	−0.012	−0.226
F-value	4.454 ***		5.991 ***		2.560 *		12.114 ***		1.957		7.839 ***		10.960 ***		8.282 ***	
R^2^	0.074		0.096		0.043		0.177		0.033		0.121		0.163		0.127	
△F	5.270 ***		6.974 ***		2.280 *		13.136 ***		2.238		9.124 ***		12.376 ***		9.631 ***	
△R^2^	0.073		0.093		0.032		0.16		0.031		0.118		0.154		0.123	

*: *p* < 0.05; **: *p* < 0.01; ***: *p* < 0.001

## References

[1] General Medical Council, Education Committee (1993). Tomorrow’s Doctor: Recommendations on Undergraduate Medical Education.

[2] Usherwood T. (1993). Subjective and behavioural evaluation of the teaching of patient interview skills. Méd. Educ..

[3] Coulter A. (1997). Partnerships with patients: The pros and cons of shared clinical decision-making. J. Health Serv. Res. Policy.

[4] Dudas R.A., Bundy D.G., Miller M.R., Barone M. (2011). Can teaching medical students to investigate medication errors change their attitudes towards patient safety?. BMJ Qual. Saf..

[5] Varjavand N., Bachegowda L.S., Gracely E., Novack D.H. (2012). Changes in intern attitudes toward medical error and disclosure. Méd. Educ..

[6] Paxton J.H., Rubinfeld I.S. (2009). Medical errors education for students of surgery: A pilot study revealing the need for action. J. Surg. Educ..

[7] Paxton J.H., Rubinfeld I.S. (2010). Medical errors education: A prospective study of a new educational tool. Am. J. Méd. Qual..

[8] Gunderson A., Tekian A., Mayer D. (2008). Teaching interprofessional health science students medical error disclosure. Méd. Educ..

[9] Gunderson A.J., Smith K.M., Mayer D.B., McDonald T., Centomani N. (2009). Teaching medical students the art of medical error full disclosure: Evaluation of a new curriculum. Teach. Learn. Med..

[10] Kaldjian L.C., Jones E.W., Wu B.J., Forman-Hoffman V.L., Levi B.H., Rosenthal G.E. (2007). Disclosing medical errors to patients: Attitudes and practices of physicians and trainees. J. Gen. Intern. Med..

[11] Martinez W., Lo B. (2008). Medical students’ experiences with medical errors: An analysis of medical student essays. Méd. Educ..

[12] Newell P., Harris S., Aufses A., Ellozy S. (2008). Student perceptions of medical errors: Incorporating an explicit professionalism curriculum in the third-year surgery clerkship. J. Surg. Educ..

[13] White A.A., Gallagher T.H., Krauss M.J., Garbutt J., Waterman A.D., Dunagan W.C., Fraser V.J., Levinson W., Larson E.B. (2008). The attitudes and experiences of trainees regarding disclosing medical errors to patients. Acad. Med..

[14] Halbach J.L., Sullivan L.L. (2005). Teaching medical students about medical errors and patient safety: Evaluation of a required curriculum. Acad. Med..

[15] Kaldjian L.C., Jones E.W., Wu B.J., Forman-Hoffman V., Levi B.H., Rosenthal G.E. (2006). Disclosing medical errors to patients: A survey of faculty, residents, and students. J. Gen. Intern. Med..

[16] Muller D., Ornstein K. (2007). Perceptions of and attitudes towards medical errors among medical trainees. Méd. Educ..

[17] Coombes I.D., Mitchell C.A., Stowasser D.A. (2008). Safe medication practice: Attitudes of medical students about to begin their intern year. Méd. Educ..

[18] (2000). Annual Report 1999.

[19] Lester H., Tritter J.Q. (2001). Medical error: A discussion of the medical construction of error and suggestions for reforms of medical education to decrease error. Méd. Educ..

[20] Seiden S., Galvan C., Lamm R. (2006). Role of medical students in preventing patient harm and enhancing patient safety. Qual. Saf. Health Care.

[21] Flin R., Yule S., McKenzie L., Paterson-Brown S., Maran N. (2006). Attitudes to teamwork and safety in the operating theatre. Surgeon.

[22] Fischer M.A., Mazor K.M., Baril J., Alper E., DeMarco D., Pugnaire M. (2006). Learning from mistakes: Factors that influence how students and residents learn from medical errors. J. Gen. Intern. Med..

[23] Lievens F., Coetsier P., De Fruyt F., De Maeseneer J. (2002). Medical students’ personality characteristics and academic performance: A five-factor model perspective. Méd. Educ..

[24] Katz-Navon T., Naveh E., Stern Z. (2007). Safety self-efficacy and safety performance: Potential antecedents and the moderation effect of standardization. Int. J. Health Care Qual. Assur..

[25] Vohra P.D., Johnson J.K., Daugherty C.K., Wen M., Barach P. (2007). Housestaff and medical student attitudes toward medical errors and adverse events. Jt. Comm. J. Qual. Patient Saf..

[26] Chang B.J. (2003). The Physician’s Knowledge, Attitude and Responses toward Patient Safety Issues—Case Studies from Hospitals in Northern Taiwan. Master’s Thesis.

[27] Robert L.H., Merritt A.C. (2017). Culture at Work in Aviation and Medicine: National, Organizational and Professional Influences.

[28] Roberts K.H., Rousseau D.M. (1989). Research in nearly failure-free, high-reliability organizations: Having the bubble. IEEE Trans. Eng. Manag..

[29] Goldberg L.R. (1992). The development of markers for the Big-Five factor structure. Psychol. Assess..

[30] Mizrahi T. (1984). Managing medical mistakes: Ideology, insularity and accountability among internists-in-training. Soc. Sci. Med..

[31] Wu A.W., Folkman S., McPhee S.J., Lo B. (1991). Do house officers learn from their mistakes?. JAMA.

[32] Schenkel S.M., Khare R.K., Rosenthal M.M., Sutcliffe K.M., Lewton E.L. (2003). Resident perceptions of medical errors in the emergency department. Acad. Emerg. Med..

[33] Hevia A., Hobgood C. (2003). Medical error during residency: To tell or not to tell. Ann. Emerg. Med..

[34] Crook E.D., Stellini M., Levine D., Wiese W., Douglas S. (2004). Medical errors and the trainee: Ethical concerns. Am. J. Med. Sci..

[35] Pilpel D., Schor R., Benbassat J. (1998). Barriers to acceptance of medical error: The case for a teaching program (695). Méd. Educ..

[36] McCrae R.R., Costa P.T. (1987). Validation of the five-factor model of personality across instruments and observers. J. Pers. Soc. Psychol..

[37] Sorokin R., Riggio J.M., Hwang C. (2005). Attitudes about patient safety: A survey of physicians-in-training. Am. J. Méd. Qual..

[38] Singh H., Thomas E.J., Petersen L.A., Studdert D.M. (2007). Medical errors involving trainees: A study of closed malpractice claims from 5 insurers. Arch. Intern. Méd..

[39] Gallagher T.H., Waterman A.D., Garbutt J.M., Kapp J.M., Chan D.K., Dunagan W.C., Fraser V.J., Levinson W. (2006). US and Canadian physicians’ attitudes and experiences regarding disclosing errors to patients. Arch. Intern. Méd..

